# Influence of the type of pathogen on the clinical course of infectious complications related to cardiac implantable electronic devices

**DOI:** 10.1038/s41598-021-94168-7

**Published:** 2021-07-21

**Authors:** Anna Polewczyk, Wojciech Jacheć, Luca Segreti, Maria Grazia Bongiorni, Andrzej Kutarski

**Affiliations:** 1 Collegium Medicum of Jan Kochanowski University, 19A, Aleja IX Wieków Kielc St., 25-317 Kielce, Poland; 2 Department of Cardiac Surgery, Świętokrzyskie Cardiology Center, Kielce, Poland; 3 2nd Department of Cardiology, Faculty of Medical Sciences in Zabrze, Medical University of Silesia, Katowice, Poland; 4 Department of Cardiology, University Hospital of Pisa, Pisa, Italy; 5 Department of Cardiology, Medical University, Lublin, Poland

**Keywords:** Microbiology, Cardiology

## Abstract

The specific role of the various pathogens causing cardiac implantable electronic devices-(CIEDs)-related infections requires further understanding. The data of 1241 patients undergoing transvenous lead extraction because of lead-related infective endocarditis (LRIE-773 patients) and pocket infection (PI-468 patients) in two high-volume centers were analyzed. Clinical course and long-term prognosis according to the pathogen were assessed. Blood and generator pocket cultures were most often positive for methicillin-sensitive *Staphylococcus aureus* (MSSA: 22.19% and 18.13% respectively), methicillin-sensitive *Staphylococcus epidermidis* (MSSE: 17.39% and 15.63%) and other staphylococci (11.59% and 6.46%). The worst long-term prognosis both in LRIE and PI subgroup was in patients with infection caused by Gram-positive microorganisms, other than staphylococci. The most common pathogens causing CIED infection are MSSA and MSSE, however, the role of other Gram-positive bacteria and Gram-negative organisms is also important. Comparable, high mortality in patients with LRIE and PI requires further studies.

## Introduction

Cardiac implantable electronic device (CIED) infection is one of the most dangerous complications in patients with pacemakers (PM), implantable cardiac defibrillators (ICD) and cardiac resynchronization therapy (CRT) devices. The rate of infections in patients with PM/ICD/CRT devices ranges between 0.5 and 2.2% that is approximately 10% of all infective endocarditis cases^1,2^. Several studies suggest that rate of infectious complications have been increasing over the last two decades and the rate of increase was disproportionate to number of implantations. Patients with PMs and ICDs showed a 2.5-fold and 1.9-fold increase in CIED infection, when comparing 1988–1994 and 1995–2001^3^. The incidence of isolated pocket infection (PI) is estimated to be 1.9/1000 device-years, whereas the incidence of pocket infection with bloodstream infection is 1.14 per 1000 device-years^4^. The most common pathogens isolated from blood and CIED pocket are staphylococci especially coagulase-negative staphylococci (CoNS) with predominantly *Staphylococcus epidermidis (S. epidermidis)* and *Staphylococcus aureus (S. aureus)*, less frequently other than staphylococci Gram-positive bacteria (the most common: streptococci, corynobacteriacae and micrococci), Gram-negative pathogens and fungi. Most available reports^5–14^ evaluate differences between CoNS and infections caused by *S. aureus.* This paper investigates the clinical and procedural factors that may influence a particular pathogen in blood and generator pocket, and analyses the impact of pathogen type on the clinical course and survival in patients undergoing transvenous lead extraction (TLE) because of infectious complications.

## Methods

### Study population and design

We retrospectively analysed data from 3810 patients undergoing TLE in two high-volume centres (Poland and Italy) between 2006 and 2017 for infectious (1758; 46.1%) and noninfectious (2052; 53.9%) indications. Because of incomplete blood and pocket culture results, we excluded 517 infectious patients from further analysis. We assessed clinical data from 1241 patients with infectious indications (773 with lead-related infective endocarditis-LRIE and 468 with isolated pocket infection-PI). The following clinical factors were taken into account: left ventricular ejection fraction (LVEF), NYHA class, prior sternotomy, presence of valvular implants, hypertension, diabetes, history of chronic kidney disease (CKD), permanent atrial fibrillation, and permanent anticoagulation or permanent antiplatelet therapy. The following TLE-related factors were analysed: device type, dwell time of the oldest lead, number of CIED-related procedures before TLE, presence of intracardiac lead abrasion, presence and size of vegetations and number of leads including the abandoned ones. We also compared LRIE and PI subgroups regarding the course and efficacy of TLE: procedure duration, technical difficulty, complete procedural and clinical success, and major and minor complications. Based on the microbiological culture results from removed leads and device pocket we compared clinical and procedural data in patients with infectious complications caused by *S. aureus* (methicillin-sensitive (MSSA) and methicillin-resistant (MRSA), *S. epidermidis* (methicillin-sensitive (MSSE) and methicillin-resistant (MRSE), other coagulase-negative staphylococci (methicillin-sensitive (MSSO) and methicillin-resistant (MRSO), other Gram-positive cocci, Gram-negative bacilli, Candida and Aspergillus (fungi), and in patients with negative blood and pocket cultures.

Using survival data obtained from the National Health Service Registry in Italy and from the National Health Fund Registry in Poland, we analysed risk factors for mortality in LRIE and PI subgroups, with particular consideration of pathogen and its impact on long-term prognosis. We constructed survival curves using the Kaplan–Meier method in a group of 3369 patients (infectious and noninfectious cases with complete follow-up).

All patients provided written informed consent prior to study enrollment. The study protocol was approved by the Bioethics Committee at the University in Lublin—ref. number 288/2018/KB/VII and complied with the Declaration of Helsinki.

### Definitions

Lead-related infective endocarditis was diagnosed using modified Duke criteria according to the 2015 ESC guidelines for the management of infective endocarditis^15^ and the European Heart Rhythm Association international consensus document^16^. The diagnosis of lead-related infective endocarditis was definite in the presence of two major criteria or one major criterion and three minor criteria. According to the current guidelines^16^, cultures from extracted leads were considered as a minor criterion in the diagnosis of LRIE. Possible LRIE was diagnosed in patients with one major and one minor criterion or 3 minor criteria. Possible LRIE was also included in the current analysis.

A pocket infection was defined as local warmth, erythema, swelling, edema, and pain in or discharge from the device pocket or an erosion or impending erosion of the device without lead-related infective endocarditis^15^.

Intracardiac lead abrasion was defined as outer insulation macroscopic damage, in the intracardiac portion of the lead, usually in the first 15–20 cm from the tip, with visible discolouration, frequently with conductor externalisation, and not infrequently with purulent discharge^17^.

Transvenous lead extraction was defined according to HRS and EHRA statements^18–20^ as a procedure involving the removal of a lead implanted for over one year or when more than just a standard stylet was required to remove the lead, or the non-implant vein approach had to be utilised. Complete procedural success of TLE was defined as removal of all targeted leads and material, with the absence of any permanently disabling complication or procedure-related death. Clinical success was achieved in patients with retention of a small part of the lead that did not negatively affect the outcome goals of the procedure^18–20^.

Major and minor complications were defined according to the 2018 EHRA Expert Consensus Statement on Lead Extraction^20^. They defined major complications as any of the outcomes related to the procedure, which was life-threatening or resulted in death or any unexpected event that caused persistent or significant disability. Minor complications were defined as any undesired event related to the procedure that required medical intervention or minor procedural intervention to remedy, and did not limit persistently or significantly the patient’s function, threaten life or cause death.

### Microbiology

In all patients with infectious complications, at least three aerobic and anaerobic blood cultures were performed before TLE. In order to exclude contamination, the criterion of a minimum of two positive cultures was used in the case of the physiological flora. In patients with the symptoms of local infection, a swab specimen was obtained from the generator pocket during TLE. The material drawn from the pocket was spread directly on the culture plate. In patients with suspected generalised infection, without PI symptoms, cultures from extracted leads were also done. A segment of the lead was rolled onto the solid media (Chocolate agar, McConkey agar, mannitol salt agar, and Sabouroud agar).

### Transvenous lead extraction

The TLE procedures in the two high-volume Polish and Italian centres were performed with the mechanical system based on cutting-rotation forces of telescopic polypropylene Byrd dilators (Cook Medical, Bloomington, IN, USA) of various lengths and sizes.

Leads were usually extracted by simple traction or mechanical dilatation using polypropylene sheaths through the implant vein. Sometimes, in more complicated cases, other venous approaches (jugular and/or femoral) or Evolution Mechanical Dilator Sheath (Cook Medical, Bloomington, IN, USA) or TightRail (Spectranetics Corporation, Colorado Springs) systems were used. We have never used laser energy or radiofrequency wave technology.

### Statistical methods

Normality was checked using the Shapiro–Wilk test, which showed that most continuous variables followed normal distribution. For uniformity, all continuous variables are presented as mean ± standard deviation and were compared using the Kruskal–Wallis ANOVA and Mann–Whitney *U* test. Categorical data are presented as absolute numbers and percentage and were compared using the Chi-square test with Yates correction.

A stepwise multivariable Cox regression model was used to evaluate the role of pathogens as risk factors for long-term mortality after TLE and included the following variables: gender, patient age at the time of TLE, NYHA class, LVEF, presence of artificial valve, arterial hypertension, diabetes mellitus, creatinine concentration, haemoglobin concentration, atrial fibrillation, anticoagulant and antiplatelet therapy, TLE of ICD leads (single or dual coil), presence of vegetations (LRIE), radiological success, pathogen type (MSSA, MRSA, MSSE, MRSE, MSSO, MRSO, other Gram-positive, Gram-negative pathogens, and fungi). Analyses were performed for patients with LRIE and pocket infection. In multivariable analysis, the results were presented as hazard ratio (HR) with 95% confidence interval (CI).

The Kaplan–Meier method was used to calculate the probability of event free survival depending on culture results from blood (LRIE) or pocket (PI) and in all subgroups divided according to indications for TLE (noninfectious, PI or LRIE). The log-rank test, including complete and censored data, was used to test for differences between survival curves.

A two-tailed *p* value < 0.05 was considered statistically significant. Statistical analysis was performed using STATISTICA 13.1 PL (TIBCO, Cracow, Poland).

## Results

**Figure 1:**
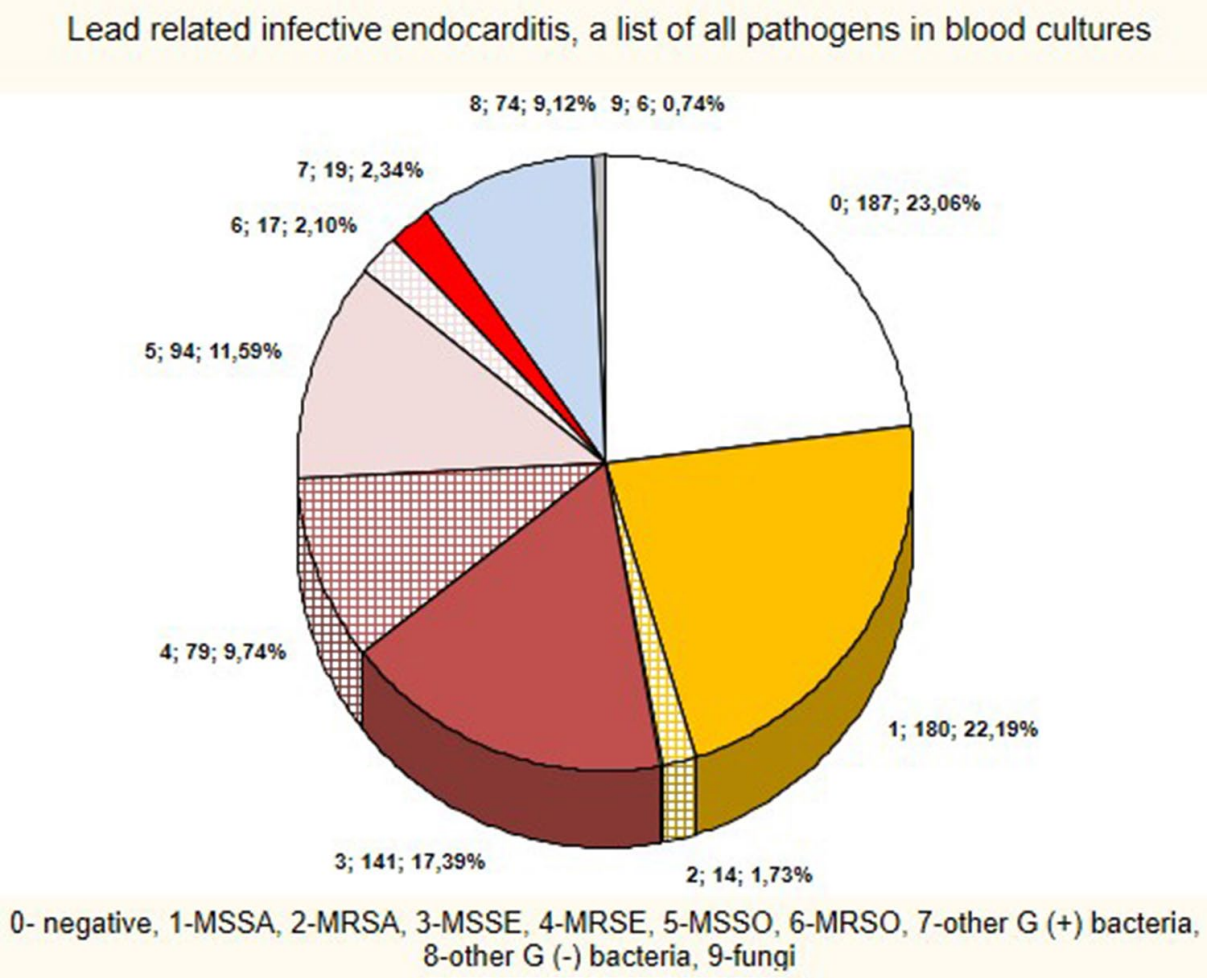
LRIE subgroup—list of pathogens.

The study population comprised 1241 patients with infectious complications, including 773 (62.29%) with LRIE, and 468 (37.71%) with isolated PI. The distribution of bacterial isolates from blood cultures in patients with LRIE was: MSSA: 22.19%, MSSE: 17.39%, MSSO: 11.59%, MRSE: 9.74%, Gram-negative: 9.12%, MRSA: 1.73%, MRSO: 2.10%, other Gram-positive: 2.34%, fungi 0.74%. Blood cultures were negative in 23.06% of cases (Fig. 1). Gram-negative were concurrent with other bacteria in 20 cases, while staphylococci were concurrent with other bacteria in 14 cases, but there was no concurrent infection of *S. aureus* or *S. epidermidis* with other pathogens (Fig. 1, Table S1).

**Figure 2:**
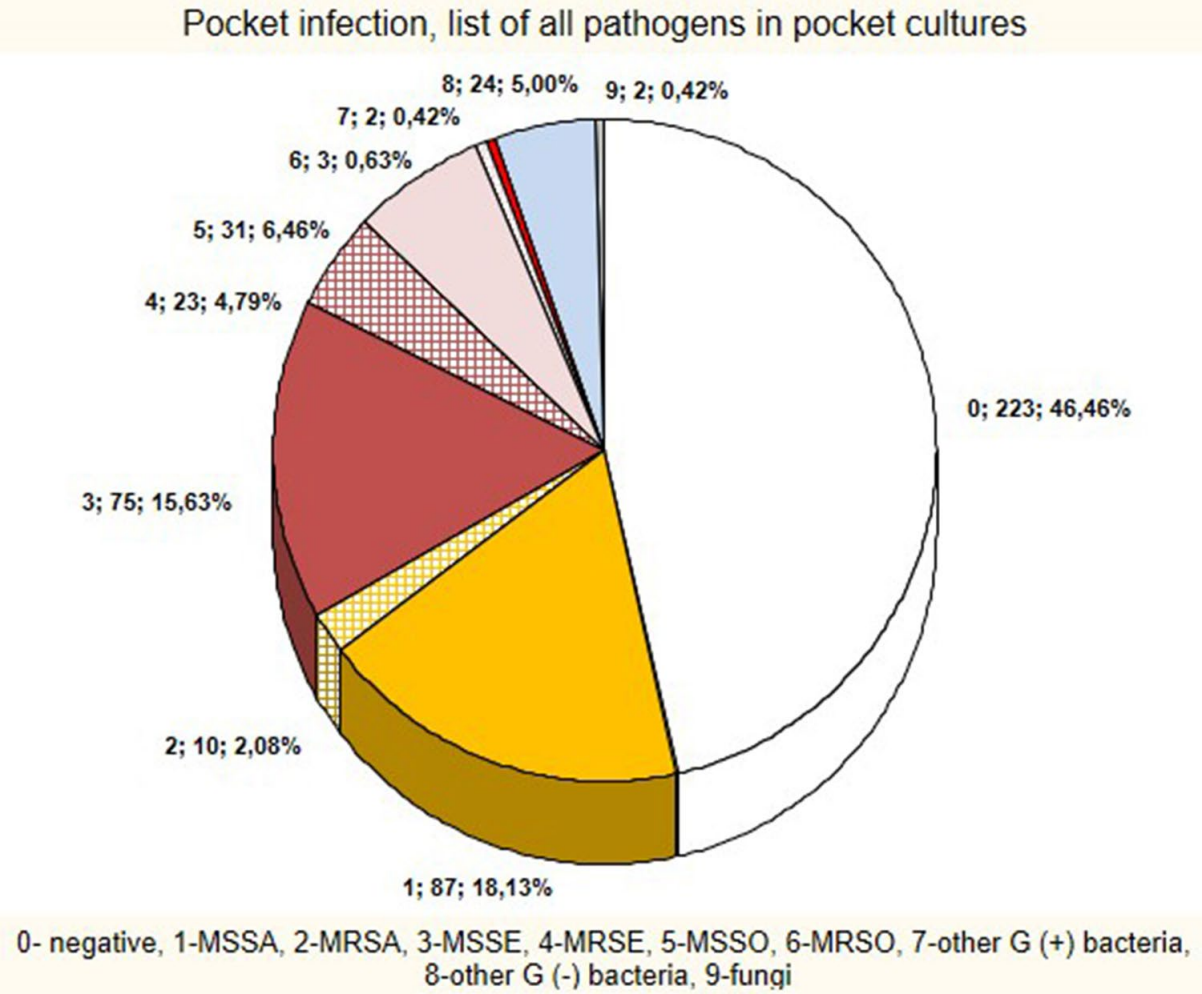
PI subgroup—list of pathogens.

In PI patients pocket cultures were positive for MSSA in 18.13%, MSSE in 15.63%, MSSO in 6.46%, Gram-negative in 5.00%, MRSE in 4.79%, MRSA in 2.08%, MRSO in 0.63%, other Gram-positive in 0.41% and fungi in 0.41%. The rate of negative cultures was 46.46% (Fig. 2). Staphylococci were concurrent with other bacteria in 5 cases, whereas Gram-negative bacteria were isolated with other pathogens in 7 patients (Fig. 2, Table S1).

In patients with suspected LRIE, besides blood cultures, 657 lead cultures from the extracted leads were analysed. Lead cultures were positive in 504 patients, including 24 patients with negative blood cultures. In these 24 patients with negative blood cultures, we identified 27 pathogens: *S. epidermidis* (51.85%) and other staphylococci (44.44%) were predominant.

### Comparison of clinical and procedural factors in patients with LRIE and PI

Patients in LRIE subgroup were more likely to have higher NYHA class (1.699 ± 0.774 vs. 1.574 ± 0.707; *p* = 0.008), diabetes mellitus (23.88% vs. 18.92%; *p* = 0.039) and renal failure (6.77% vs. 3.15%; *p* = 0.039), but permanent atrial fibrillation (21.76% vs. 26.82%; *p* = 0.042) was less common than in PI patients. The remaining clinical parameters were similar in both subgroups (Table 1).

**Table 1:** Demographic and clinical data of patients admitted for lead extraction due to infection.

	All group (patients with CIED related infections) N = 1241	LRIE N = 773 (62.29)	PI N = 468 (37.71)	*p* “U” Mann–Whitney/Chi2
**Demographics**				
Male n (%)	908 (73.00) n = 1241	559 (72.32) n = 773	349 (74.15) n = 468	*p* = 0.389
Female n (%)	333 (26.99) n = 1241	214 (27.68) n = 773	119 (25.85) n = 468	*p* = 0.389
Mean age (first implantation) mean ± sd	60.811 ± 14.966 n = 1241	60.435 ± 14.693 n = 773	61.365 ± 15.278 n = 468	0.107
Mean age (TLE), mean, SD Mean ± SD	67.947 ± 13.662 n = 1241	67.802 ± 13.378 n = 773	68.130 ± 14.053 n = 468	0.380
BMI [kg/m^2^] Mean ± SD	27.105 ± 4.028 n = 1199	27.205 ± 4.252 n = 710	26.917 ± 3.583 n = 389	0.250
**Clinical data**				
LVEF [%] Mean ± SD	48.114 ± 14.363 n = 1190	48.107 ± 14.226 n = 750	48.050 ± 14.249 n = 440	*p* = 0.806
NYHA class Mean ± SD	1.740 ± 3.143 n = 1235	1.699 ± 0.774 n = 770	1.574 ± 0.707 n = 465	0.008
Presence of valvular implants n (%)	75 (6.04) n = 1241	46 (5.95) n = 773	29 (6.20) n = 468	*p* = 0.857
Prior sternotomy n (%)	194 (15.84) n = 1225	122 (15.80) n = 759	72 (16.14) n = 466	*p* = 0.881
Arterial hypertension n (%)	774 (62.37) 1241	486 (62.87) 773	288 (61.54) 468	*p* = 0.682
Diabetes n (%)	270 (22.00) n = 1227	182 (23.88) n = 762	88 (18.92) n = 465	*p* = 0.039
History of CKD or eGFR < 60 ml/min/1.73 m^2^ n (%)	62 (5.45) n = 1137	49 (6.77) n = 724	13 (3.15) n = 413	*p* = 0.014
Creatinine concentrations [mg/dl] Mean ± SD	1.264 ± 0.831 n = 1137	1.308 ± 0.865 n = 724	1.186 ± 0.762 n = 413	*p* = 0.021
Permanent atrial fibrillation n (%)	291 (23.68) n = 1229	166 (21.76) n = 763	125 (26.82) n = 466	*p* = 0.042
Permanent anticoagulation n (%)	391 (32.58) n = 1200	248 (32.98) n = 752	144 (32.14) n = 448	*p* = 0.810
Permanent antiplatelet therapy n (%)	476 (39.67) n = 1200	304 (40.43) n = 752	172 (38.39) n = 448	*p* = 0.447
**Preoperative pacing system information**				
Device without ICD lead n (%)	794 (63.98) n = 1241	499 (64.55) n = 773	295 (63.03) n = 468	*p* = 0.550
Device with ICD lead n (%)	447 (36.02) 1241	274 (35.45) 773	173 (36.97) 468	*p* = 0.550
Dwelling time for oldest lead [months], Mean ± SD	86.525 ± 69.373 n = 1241	89.187 ± 69.686 n = 773	82.011 ± 68.717 n = 468	*p* = 0.047
Number of CIED-related operative procedures before TLE (upgrading, pocket revision) Mean ± SD	0.838 ± 1.197 n = 1241	0.816 ± 1.266 n = 773	0.868 ± 1.065 n = 468	*p* = 0.016
Number of all operative procedures before TLE (implantations, reimplantation, upgrading, pocket revision) Mean ± SD	2.413 ± 1.561 n = 1241	2.425 ± 1.654 n = 773	2.385 ± 1.389 n = 468	*p* = 0.306
Presence of intracardiac lead abrasion n (%)	213 (26.93) n = 791	163 (29.37) n = 555	50 (21.19) (n = 236)	*p* = 0.017
Number of leads in system Mean ± SD	1.956 ± 0.673 n = 1241	1.948 ± 0.670 n = 773	1.970 ± 0.679 n = 468	*p* = 0.559
Number of patients with abandoned leads n (%)	246 (19.82) N = 1241	162 (20.95) N = 773	84 (17.95) N = 468	*p* = 0.224
Mean number of abandoned leads in patients Mean ± SD	0.255 ± 0.627 n = 1241	0.267 ± 0.613 n = 773	0.235 ± 0.639 n = 468	*p* = 0.304
Total number of leads in patient Mean ± SD	2.211 ± 0.859 n = 1241	2.214 ± 0.873 n = 773	2.205 ± 0.820 n = 468	*p* = 0.889
Vegetations presence n (%)	522 (42.06) n = 1241	522 (67.53) n = 773	0 (0.00) n = 468	*p* < 0.001
Presence of large vegetation > 2 cm^2^ n (%)	146 (11.76) n = 1241	146 (18.89) n = 773	0 (0.00) n = 468	*p* < 0.001
**TLE procedure**				
Technical problems or complications during TLE Mean ± SD	0.152 ± 0.359 n = 1241	0.145 ± 0.352 n = 773	0.163 ± 0.370 n = 468	*p* = 0.401
Number of patients with technical problems or complications during TLE n(%)	188 (15.15) n = 1241	112 (14.45) n = 773	76 (16.24) n = 468	*p* = 0.401
Procedure duration time [min] Mean ± SD	105.280 ± 50.684 n = 1241	107.854 ± 50.017 n = 766	100.869 ± 44.346 n = 464	*p* = 0.077
Radiological success n (%)	1172 (94.44) n = 1241	732 (94.70) n = 773	441 (94.23) n = 468	*p* = 0.254
Clinical success n (%)	1199 (96.62) n = 1241	749 (96.90) n = 773	450 (96.15) n = 468	*p* = 0.594
Major complications n (%)	18 (1.45) n = 1241	14 (1.81) n = 773	4 (0.85) n = 468	*p* = 0.175
Minor complications n (%)	69 (5.56) n = 1241	48 (6.21) n = 773	21 (4.49) n = 468	*p* = 0.200
Periprocedural deaths n (%)	7 (5.46) n = 1241	6 (0.78) n = 773	1 (0.21) n = 468	*p* = 0.200
Follow-up [days] Mean ± SD	1589,0 ± 1041,2 n = 1071	1543,9 ± 1042,1 n = 696	1672,7 ± 1035,7 n = 375	*p* = 0.732
Death during follow-up n (%)	369 (34.45) n = 1071)	254 (36.49) n = 696)	115 (30.67) n = 375	*p* = 0.065

AF, atrial fibrillation; CIED, cardiac implantable electronic devices; CKD, chronic kidney disease; eGFR, glomerular filtration rate; ICD, implantable cardioverter defibrillator; LRIE, lead related infective endocarditis, LVEF, left ventricular ejection fraction, PI, pocket infections, SD, standard deviation, TLE, transvenous lead extraction.

About device-related and procedural factors, patients with LRIE had older leads (89.187 ± 69.686 vs. 82.011 ± 68.717 dwelling time, in months; *p* = 0.047) and more frequent intracardiac lead abrasion (29.37% vs. 21.19%; *p* = 0.017), whereas patients with PI underwent more CIED-related procedures (upgrading, revision of the pocket) (0.868 ± 1.065 vs. 0.816 ± 1.266; *p* = 0.016). The remaining factors that might influence the course of TLE did not differ between LRIE and PI subgroups. Course of the extraction procedure, radiological and clinical success rates, technical complications and major and minor complications, and mortality during follow-up did not differ between subgroups (Table 1).

### LRIE patients: analysis of clinical and procedural factors according to pathogens isolated from blood

Among patients with LRIE divided according to pathogen type, males were most likely to have staphylococcal infection (especially *S. aureus*; 82.99%). Patients with infection caused by *S. aureus* were the oldest at the time of first device implantation (mean age 63.902 ± 13.664 years), whereas patients with *S. epidermidis* and with other staphylococci in blood cultures were the youngest at implantation (58.973 ± 14.707 and 58.320 ± 15.184 years, respectively). Mean age of all patients with LRIE during TLE was similar, regardless of the pathogen type. Patients with infection caused by *S. aureus* were most likely to have chronic kidney disease, whereas in patients with *S. epidermidis* and other staphylococci, CKD was less common. Patients with Gram-negative bacteria in blood cultures were more often on chronic anticoagulation (51.92%) as compared with other subgroups. The remaining clinical parameters were similar for all pathogens (Table S2).

On device-related factors, patients with infection caused by *S. aureus* had shorter lead dwell times (dwell time of the oldest extracted lead was 75.954 ± 70.027 months) whereas patients with other staphylococci and *S. epidermidis* had the oldest leads (lead dwell times of 98.711 ± 67.54 and 93.740 ± 65.033 months, respectively).

The highest number of CIED-related procedures before TLE (2.785 ± 1.778) characterised patients with infection caused by *S. epidermidis*. Patients with *Candida/Aspergillus* and other staphylococci had the least number of leads in the system (1.333 ± 0.516 and 1.814 ± 0.565, respectively). Rates of complete procedural success, clinical success, major and minor complications were similar in all LRIE subgroups. Vegetations were most common in patients with infections caused by *S. aureus* (67.01%) (Table S2).

### PI patients: analysis of clinical and procedural factors according to pathogens isolated from generator pocket

*S. epidermidis* (20.94%), *S. aureus* (20.73%) and other staphylococci (6.62%) were the predominant pathogens in patients with PI, while other Gram-positive bacteria (0.43%) were the least common organisms. *S. aureus* was most present in males (83.51%), since Gram-negative bacteria were more common in women (64.71%). Patients with other Gram-positive bacteria in pocket cultures were the oldest (80.000 ± 4.243 years), whereas patients with other staphylococci were the youngest (59.323 ± 17.213 years) at the time of device implantation. The subgroups did not differ in patient age during TLE. There were no subgroup differences in left ventricular ejection fraction, but patients with other Gram-positive bacteria in cultures were in the highest NYHA class (3.000 ± 0.000 on average), whereas the lowest NYHA class was observed in patients with other staphylococci (1.355 ± 0.551). Gram-negative bacteria in pocket cultures were more common in patients after sternotomy (41.18% patients). The remaining clinical factors did not differ between subgroups (Table S3).

Among procedural factors, lead dwell times were shorter in patients with local infection because of Gram-negative bacteria (41.412 ± 44.626 months). Patients with other staphylococci had a higher number of implanted leads (2.226 ± 0.735) as compared with *S. aureus* and *S. epidermidis* subgroups. Abandoned leads were most common in *S. aureus* subgroup i.e. in 23.71% of patients. The efficacy of TLE and major and minor complications were similar in all subgroups (Table S3).

### Long-term survival after TLE: analysis according to type of infectious complications

**Figure 3:**
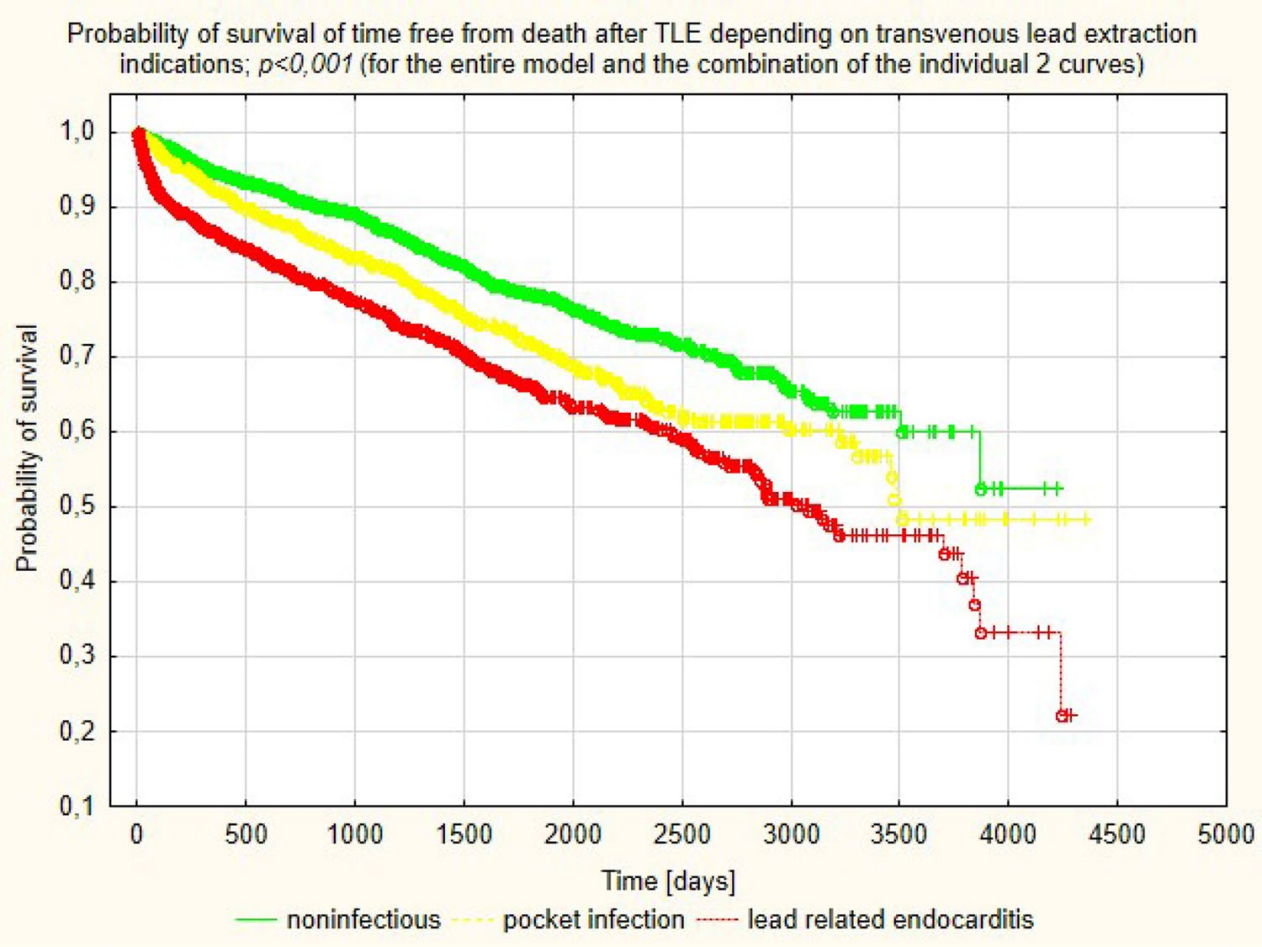
Kaplan–Meier probability of survival depending on indications for transvenous lead extraction procedure.

During long-term follow-up (median: 4.121 years; IQR 2.041–6.648) there were 254 (36.49%) deaths in LRIE subgroup and 115 (30.67%) deaths in PI patients (*p* = 0.065) (Table 1). Mortality in patients with LRIE and PI was significantly higher than in patients undergoing TLE for noninfectious indications (*p* < 0.001) (Fig. 3).

### Long term survival after TLE in LRIE patients: analysis according to pathogen

**Figure 4:**
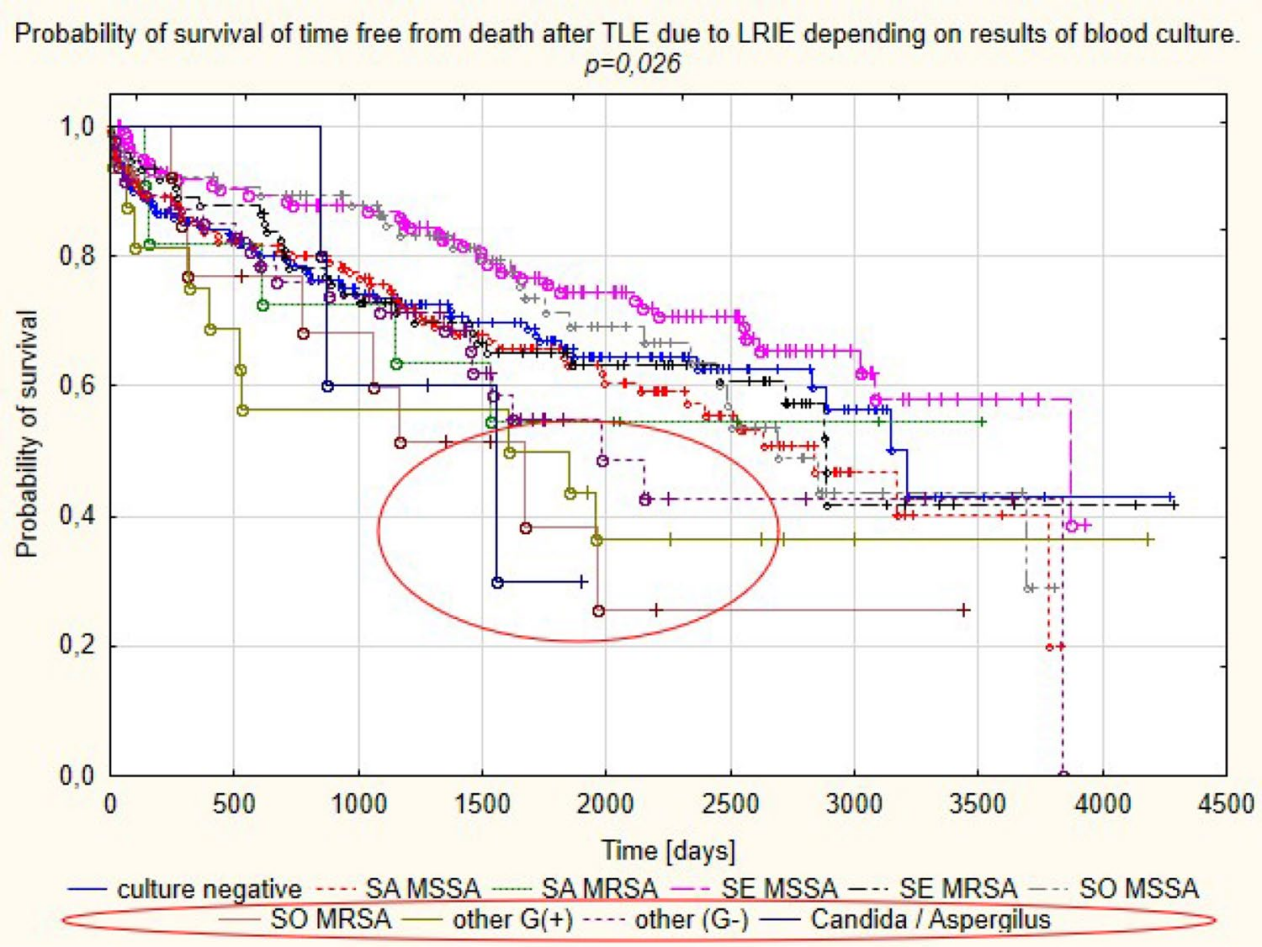
Probability of survival in patients with LRIE depending on pathogen type.

At long-term follow-up the highest mortality was observed in patients with other than staphylococci Gram-positive bacteria (62.5% of deaths during mean follow-up of 1503.8 ± 1265.8 days) and with Gram-negative bacteria (mortality of 43.75% during mean follow-up of 1291.2 ± 450.0 days). Fungal infection was associated with high mortality rates (60% of deaths during mean follow-up of 1291.2 ± 450.0 days) during a short follow-up. The lowest mortality rate was observed in patients with negative blood cultures (32.56% during 1368.3 ± 1024.8 days of follow-up) and with *S. epidermidis* infection (34.33% of deaths during mean follow-up of 1867.4 ± 1044 days) (Table S2, Fig. 4).

### Long term survival after TLE in LRIE patients: Multivariable analysis of mortality risk factors

Factors that increased mortality risk during long-term follow-up were heart failure (higher NYHA class and lower LVEF), permanent atrial fibrillation, renal failure (higher creatinine levels), anaemia and older patient age. Infection due to MSSE was associated with better long-term survival rates (Table 2).

**Table 2:** Risk factors for deaths after TLE in LRIE patients.

n = 641 (complete data)	HR	95% CI	*p*
Age of patients during TLE (1 year)	1.031	1.019–1.044	< 0.001
NYHA (1 class)	1.500	1.250–1.800	< 0.001
Lower LVEF (1%p)	1.017	1.007–1.027	< 0.001
Permanent atrial fibrillation	1.389	1.050–1.837	0.021
Lower haemoglobin concentration (1 g/dl)	1.075	1.005–1.149	0.035
Creatinine (1 mg %)	1.277	1.134–1.439	< 0.001
Pathogen other than MSSE	1.876	1.256–2.703	< 0.001

Results of multivariable stepwise Cox regression model.

LRIE, lead related infection endocarditis, LVEF, left ventricular ejection fraction, MSSE, methicillin sensitive *S. epidermidis*; NYHA, New York Heart Association (class), TLE, transvenous lead extraction.

### Long term survival after TLE in PI patients: analysis according to pathogen

**Figure 5:**
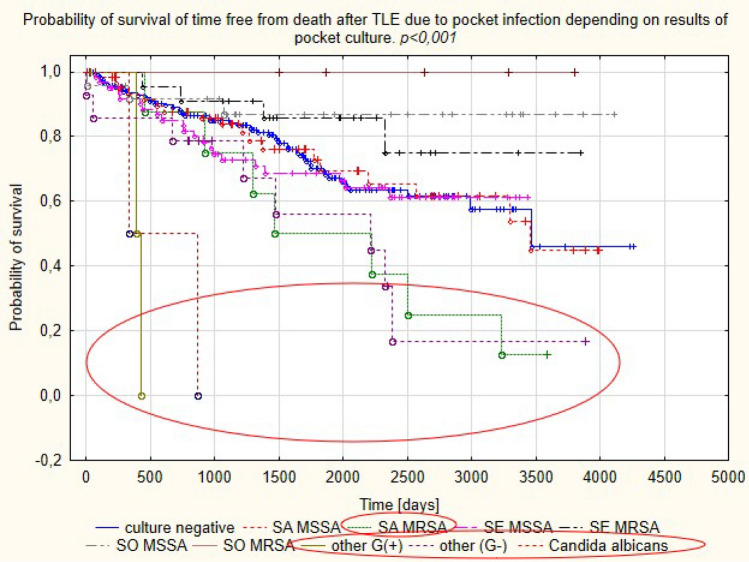
Probability of survival in patients with PI depending on pathogen type.

During follow-up of 407.00 ± 26.87 days, the highest mortality (100%) was observed in patients with *Candida albicans* (mean follow-up of 602.00 ± 374.767 days) and other than staphylococci Gram-positive bacteria in pocket cultures. Death rates were also high in patients with Gram-negative bacteria in pocket cultures (57.14% of deaths during mean follow-up of 1430.5 ± 1077.1 days). We observed best survival outcomes in patients with other than *S. aureus* and *S. epidermidis* organisms in pocket cultures (13.3% mortality during mean follow-up of 2091.1 ± 1221.3 days) (Table S3, Fig. 5).

### Long-term survival after TLE in PI patients: multivariable analysis of mortality risk factors

At long-term follow-up *Candida albicans* isolated from pocket cultures, diabetes, heart failure (higher NYHA class and lower LVEF), arterial hypertension, anaemia and older patient age were the risk factors for mortality in patients with PI (Table 3).

**Table 3:** Risk factors for deaths after TLE in PI patients.

n = 325 (complete data)	HR	95% CI	*p*
Age of patients during TLE (1 year)	1.023	1.003–1.043	0.022
NYHA (1 class)	1.705	1.251–2.324	< 0.001
Lower LVEF (1%p)	1.018	1.003–1.034	0.018
Arterial hypertension	1.671	1.019–2.738	0.042
Diabetes	1.809	1.136–2.879	0.012
Lower haemoglobin concentration (1 g/dl)	1.311	1.166–1.473	< 0.001
Creatinine (1 mg %)	1.144	0.992–1.319	0.065
*Candida albicans* pocket culture	15.630	3.451–70.798	< 0.001

Results of multivariable stepwise Cox regression model.

LVEF—Left ventricular ejection fraction; NYHA—New York Heart Association (class); PI—pocket infection; TLE—transvenous lead extraction.

## Discussion

Infectious complications in patients with CIEDs have specific characteristics related to the foreign body reaction. There are two mechanisms of CIED infection. One mechanism involves contamination at the time of first implantation or other CIED-related procedures before TLE, with subsequent bacterial colonisation and infection spread along the leads to the endocardium. The other mechanism is associated with primary bloodstream infection^4,21^. Infectious complications in CIED patients can be divided into local pocket infection and a generalised response i.e. lead-related infective endocarditis. Comparative analysis of patients with PI and LRIE in this study, similar to earlier reports^22–24^, showed that patients with generalised infection were most likely to have concomitant diseases (heart failure, diabetes, renal failure, permanent atrial fibrillation), and specific procedural factors: longer lead dwell times and intracardiac lead abrasions. PI, as compared with LRIE, was associated with a higher number of CIED-related procedures before TLE. Detailed microbiological analysis in the study population confirmed that MSSA and MSSE were the most frequent pathogens, causing infectious complications in patients with CIEDs. The incidence of these organisms has been reported to range between 17 and 45% and between 20.5 and 67%, which is comparable to that in the present study^5–14^. Our analysis also revealed a relatively frequent (11.59%) incidence of MSSO, with coagulase-negative strains such as *S. hominis, S. capitis, S. haemolyticus* being predominant. Isolation of CoNS usually represents a challenge for the clinician since it may result from contamination, given the generally low pathogenicity of CoNS except for *S. lugdunensis* which causes infections with a more severe course, resembling that of *S. aureus*^8^. These considerations may explain the different outcome of patients with isolation of other staphylococci in case of PI or LRIE (Figs. 4, 5).

Similar to earlier studies^10,14,25^ this investigation confirmed the observations on risk factors and course of infections caused by *S. aureus* and other CoNS. *S. aureus* infection occurred in patients with shorter lead dwell times, whereas infection caused by *S. epidermidis* was more common in patients with earlier CIED-related procedures.

Apart from the most typical staphylococcal etiology in CIED infection, in this study Gram-negative bacteria caused infection in 9.12% of LRIE patients and in 5% of PI patients. Similar levels of incidence rates have been reported in recent years^26,27^. According to earlier studies, Gram-negative bacteria were rarely the primary cause of CIED infection, a milder course and lower long-term mortality characterised which, as compared with typical staphylococcal infection^28^. However, recent reports have showed that CIED is one of significant risk factors for the development of infective endocarditis because of Gram-negative organisms^26^. This study showed that Gram-negative bacteria was associated with a high rate of death among patients with LRIE and PI (43.75% and 57.14%, respectively) during 4-year follow-up after TLE. Observations about long-term prognosis in patients with CIED infection due to Gram-negative bacteria are very rare. Recent studies in populations with infective endocarditis including LRIE have shown the 15%-30% mortality rate at one-year follow-up^26,29^. The worse survival rate of patients with Gram negative infections in the current population may be related to the relatively high coexistence of other bacteria in cultures.

Analysis of long-term survival data from patients undergoing TLE because of CIED infection in our study showed the highest death rates in patients with other than staphylococci Gram positive infections (62.5% in LRIE and 100% in PI subgroup). This pathogenic group has first streptococci, corynobacteriacae and micrococci. The subgroup size in our study was small (16 patients with LRIE and 2 patients with PI), but these results are consistent with other research which found high mortality in patients with less typical CIED infection, caused by streptococci^25,30^. Detailed microbiological analysis of CIED infections in this study showed also best long-term prognosis for patients with MSSE in blood cultures. These findings match those observed in earlier studies which compared first survival in patients with *S. aureus* and CoNS^14^. Most of the studies focused on in-hospital and 12-month mortality showing that *S. aureus* infection was associated with about 20–25% of deaths at one year and the shortest survival^14,30,31^. It should be emphasised that there are only single reports assessing mortality over a longer follow-up period. Analysis of survival in 5817 patients with CIED infection showed a 53.8% rate of death in patients after PM implantation during 3-year follow-up vs. 33% in patients without infection (*p* < 0.001), 47.7% vs. 31.6% (*p* < 0.001) in patients with ICDs, and 50.8% vs. 36.5% (*p* < 0.001) in patients with CRT devices. The investigators emphasise that the cause of such high mortality rates is unknown and requires further research^32^. In contrast, a study in 197 patients with CIED infection showed a 35.4% mortality rate at 5 years (mean follow-up 25 months (12–70) and the rate was like that in the noninfectious control group^9^. Our study during mean follow-up of 1589.0 ± 1041.2 days showed a 36.49% rate of death in patients with LRIE and 30.67% in patients with PI, which was higher than compared with death rates in patients undergoing TLE for noninfectious reasons. Although direct comparison showed that differences in death rates between PI and LRIE subgroups approached borderline statistical significance (Chi2 *p* = 0.065), survival curves differed between subgroups (log rank *p* < 0.001). Most of the available studies show better survival in patients with local infection than in those with generalised infection^30,33,34^. A recent study has documented similar survival rates in patients with PI and LRIE during over 2-year follow-up (*p* = 0.791), but the study was carried out in a small group of patients (107 subjects)^35^. We need further research to explain reasons behind poor long-term survival in PI patients in our study; this result can be attributed to difficulties in evaluating infection spread. This is likely because the present study documented high mortality in the PI and LRIE subgroups with the same cultures: other than Gram-positive and Gram-negative bacteria. Another important issue is the try to explain the reasons for the worse long-term survival in patients after treatment because of CIED infection. Both in LRIE and PI patients the predominant risk factors for mortality were clinical factors: patient age, heart failure, atrial fibrillation, renal failure, anaemia. In PI subgroup it was arterial hypertension, diabetes and local infection with *Candida albicans*. Several reports have shown a similar relationship between clinical factors and long-term survival after CIED infection^9,30,34,36,37^, but detailed analysis of risk adjusted for age, gender, race/ethnicity, and the 28 comorbidities from the Elixhauser system in a large population of PM/ICD/CRT-D recipients^32^ showed that high mortality in patients with CIED infection can persist for at least 1–3 years after the infection and is device-dependent. The worse long-term prognosis in patients after treatment for CIED infection prompts us to continue looking for the causes of low efficacy of the therapy.

## Study limitations

This is a retrospective analysis, and we collected the data only in two high-volume centres of TLE. Because of this limitation it is not possible to assess the causes of the high rates of negative blood and pocket cultures, which is probably related to sampling during antibiotic therapy in institutions referring patients for TLE. There is no possibility of evaluating the post-TLE course (as we refer patients to the sending institution)–duration of antibiotic treatment, which may affect long-term survival, is unknown. It is not possible, either, to test the direct causes of death during long-term follow-up.

## Conclusions

CIED infection contributes to a poorer clinical course and long-term prognosis in patients undergoing transvenous lead extraction. The most common pathogens causing CIED infection are MSSA and MSSE, however special attention should be given to the adverse effects on the long-term survival in patients with other Gram-positive bacteria and Gram-negative organisms in blood or pocket cultures. Clinical assessment of patients with local infection is important, as the long-term prognosis in this group is comparable to that in LRIE patients.

## Supplementary Information

Supplementary Tables.

## Abbreviations

CIED: Cardiac implantable electronic device
CoNS: Coagulase-negative staphylococci
CKD: Chronic kidney disease
CRT: Cardiac resynchronization therapy
ICD: Implantable cardiac defibrillators
LRIE: Lead related infective endocarditis
LVEF: Left ventricle ejection fraction
MSSA: Methicillin sensitive *Staphylococcus aureus*
MRSA: Methicillin resistant *Staphylococcus aureus*
MSSO: Methicillin sensitive other staphylococcus
MRSA: Methicillin resistant other staphylococcus
NYHA: New York Heart Association class
TLE: Transvenous lead extraction

## References

[1] Sandoe JA, Barlow G, Chambers JB, Gammage M, Guleri A, Howard P, Olson E, Perry JD, Prendergast BD, Spry MJ, Steeds RP, Tayebjee MH, Watkin R (2015). British guidelines for the diagnosis, prevention and management of implantable cardiac electronic device infection. J Antimicrob Chemother.

[2] Murdoch DR, Corey GR, Hoen B, Miró JM, Fowler VG, Bayer AS, Karchmer AW, Olaison L, Pappas PA, Moreillon P, Chambers ST, Chu VH, Falcó V, Holland DJ, Jones P, Klein JL, Raymond NJ, Read KM, Tripodi MF, Utili R, Wang A, Woods CW, Cabell CH, International Collaboration on Endocarditis-Prospective Cohort Study (ICE-PCS) Investigators (2009). Clinical presentation, etiology, and outcome of infective endocarditis in the 21st century: The International Collaboration on Endocarditis-Prospective Cohort Study. Arch Intern Med..

[3] Dai M, Cai C, Vaibhav V, Sohail MR, Hayes DL, Hodge DO, Tian Y, Asirvatham R, Cochuyt JJ, Huang C, Friedman PA, Cha YM (2019). Trends of cardiovascular implantable electronic device infection in 3 decades: A population-based study. JACC Clin Electrophysiol..

[4] Uslan DZ, Sohail MR, St Sauver JL, Friedman PA, Hayes DL, Stoner SM, Wilson WR, Steckelberg JM, Baddour LM (2007). Permanent pacemaker and implantable cardioverter defibrillator infection: A population-based study. Arch Intern Med.

[5] Sohail MR, Uslan DZ, Khan AH, Friedman PA, Hayes DL, Wilson WR, Steckelberg JM, Stoner S, Baddour LM (2007). Management and outcome of permanent pacemaker and implantable cardioverter–defibrillator infections. J Am Coll Cardiol..

[6] Sohail MR, Uslan DZ, Khan AH, Friedman PA, Hayes DL (2008). Infective endocarditis complicating permanent pacemaker and implantable cardioverter–defibrillator infection. Mayo Clin Proc.

[7] Massoure PL, Reuter S, Lafitte S, Laborderie J, Bordachard P, Clementy J, Roudaut R (2007). Pacemaker endocarditis: clinical features and management of 60 consecutive cases. Pacing Clin Electrophysiol.

[8] Bongiorni MG, Tascini C, Tagliaferri E, Di Cori A, Soldati E, Leonildi A, Zucchelli G, Ciullo I, Menichetti F (2012). Microbiology of cardiac implantable electronic device infections. Europace.

[9] Deharo JC, Quatre A, Mancini J, Khairy P, Le Dolley Y, Casalta JP, Peyrouse E, Prévôt S, Thuny F, Collart F, Raoult D, Habib G, Franceschi F (2012). Long-term outcomes following infection of cardiac implantable electronic devices: A prospective matched cohort study. Heart.

[10] Greenspon AJ, Prutkin JM, Sohail MR, Vikram HR, Baddour LM, Danik SB, Peacock J, Falces C, Miro JM, Blank E, Naber C, Carrillo RG, Tseng CH, Uslan DZ (2012). Timing of the most recent device procedure influences the clinical outcome of lead-associated endocarditis results of the MEDIC (Multicenter Electrophysiologic Device Infection Cohort). J Am Coll Cardiol..

[11] Gandhi T, Crawford T, Riddell J (2012). Cardiovascular implantable electronic device associated infections. Infect Dis Clin N Am..

[12] Tarakji KG, Chan EJ, Cantillon DJ, Doonan AL, Hu T, Schmitt S, Fraser TG, Kim A, Gordon SM, Wilkoff BL (2010). Cardiac implantable electronic device infections: Presentation, management, and patient outcomes. Heart Rhythm.

[13] Viola GM, Awan LL, Ostrosky-Zeichner L, Chan W, Darouiche RO (2012). Infections of cardiac implantable electronic devices a retrospective multicenter observational study. Medicine.

[14] Le KY, Sohail MR, Friedman PA, Uslan DZ, Cha SS, Hayes DL, Wilson WR, Steckelberg JM, Baddour LM, Mayo Cardiovascular Infections Study Group (2012). Clinical features and outcomes of cardiovascular implantable electronic device infections due to Staphylococcal species. Am J Cardiol..

[15] Habib G, Lancellotti P, Antunes MJ, Bongiorni MG, Casalta JP, Del Zotti F, Dulgheru R, El Khoury G, Erba PA, Iung B, Miro JM, Mulder BJ, Plonska-Gosciniak E, Price S, Roos-Hesselink J, Snygg-Martin U, Thuny F, Tornos Mas P, Vilacosta I, Zamorano JL, ESC Scientific Document Group (2015). ESC guidelines for the management of infective endocarditis: the Task Force for the Management of Infective Endocarditis of the European Society of Cardiology (ESC). Endorsed by: European Association for Cardio-Thoracic Surgery (EACTS), the European Association of Nuclear Medicine (EANM). Eur Heart J.

[16] Blomström-Lundqvist C, Traykov V, Erba PA, Burri H, Nielsen JC, Bongiorni MG, Poole J, Boriani G, Costa R, Deharo JC, Epstein LM, Sághy L, Snygg-Martin U, Starck C, Tascini C, Strathmore N. European Heart Rhythm Association (EHRA) international consensus document on how to prevent, diagnose, and treat cardiac implantable electronic device infections-endorsed by the Heart Rhythm Society (HRS), the Asia Pacific Heart Rhythm Society (APHRS), the Latin American Heart Rhythm Society (LAHRS), International Society for Cardiovascular Infectious Diseases (ISCVID), and the European Society of Clinical Microbiology and Infectious Diseases (ESCMID) in collaboration with the European Association for Cardio-Thoracic Surgery (EACTS) Eur Heart J. 2020; 1; 41: 2012–2032 10.1093/eurheartj/ehaa010 32101604

[17] Kutarski A, Małecka B, Kołodzinska A, Grabowski M (2013). Mutual abrasion of endocardial leads: Analysis of explanted leads. Pacing Clin Electrophysiol..

[18] Wilkoff BL, Love CJ, Byrd CL, Bongiorni MG, Carrillo RG, Crossley GH 3rd, Epstein LM, Friedman RA, Kennergren CE, Mitkowski P, Schaerf RH, Wazni OM (2009) Heart Rhythm Society; American Heart Association: Transvenous Lead Extraction: Heart Rhythm Society Expert Consensus on Facilities, Training, Indications, and Patient Management. Heart Rhythm, 2009; 6: 1085–1104. 10.1016/j.hrthm.2009.05.020 19560098

[19] Kusumoto FM, Schoenfeld MH, Wilkoff BL, Berul CI, Birgersdotter-Green UM, Carrillo R, Cha YM, Clancy J, Deharo JC, Ellenbogen KA, Exner D, Hussein AA, Kennergren C, Krahn A, Lee R, Love CJ, Madden RA, Mazzetti HA, Moore JC, Parsonnet J, Patton KK, Rozner MA, Selzman KA, Shoda M, Srivathsan K, Strathmore NF, Swerdlow CD, Tompkins C, Wazni O (2017). 2017 HRS expert consensus statement on cardiovascular implantable electronic device lead management and extraction. Heart Rhythm.

[20] Bongiorni MG, Burri H, Deharo JC, Starck C, Kennergren C, Saghy L, Rao A, Tascini C, Lever N, Kutarski A, Fernandez Lozano I, Strathmore N, Costa R, Epstein L, Love C, Blomstrom-Lundqvist C; ESC Scientific Document Group. ESC Scientific Document Group. 2018 EHRA expert consensus statement on lead extraction: Recommendations on definitions, endpoints, research trial design, and data collection requirements for clinical scientific studies and registries: Endorsed by APHRS/HRS/LAHRS. Europace. 2018 1; 20: 1217 10.1093/europace/euy050 29566158

[21] Da Costa A, Lelièvre H, Pha D, Kirkorian G, Célard M, Chevalier P, Vandenesch F, Etienne J, Touboul P (1998). Role of the preaxillary flora in pacemaker infections. Circulation.

[22] Golzio PG, Fanelli AL, Vinci M, Pelissero E, Morello M, Marra WG, Gaita F (2013). Lead vegetations in patients with local and systemic cardiac device infections: Prevalence, risk factors, and therapeutic effects. Europace.

[23] Ipek EG, Guray U, Demirkan B, Guray Y, Aksu T (2012). Infections of implantable cardiac rhythm devices: Predisposing factors and outcome. Acta Cardiol..

[24] Polewczyk A, Jacheć W, Janion M, Podlaski R, Kutarski A (2015). Lead-dependent infective endocarditis: The role of factors predisposing to its development in an analysis of 414 clinical cases. Pacing Clin. Electrophysiol..

[25] Madhavan M, Sohail MR, Friedman PA, Hayes DL, Steckelberg JM, Wilson WR, Baddour LM; Mayo Cardiovascular Infections Study Group. Outcomes in patients with cardiovascular implantable electronic devices and bacteremia caused by Gram-positive cocci other than *Staphylococcus aureus*. Circ Arrhythm Electrophysiol. 2010; 3: 639–645 10.1161/CIRCEP.110.957514 20852296

[26] Falcone M, Tiseo G, Durante-Mangoni E, Ravasio V, Barbaro F, Ursi MP, Pasticci MB, Bassetti M, Grossi P, Venditti M, Rizzi M (2018). Risk factors and outcomes of endocarditis due to non-HACEK gram-negative bacilli: Data from the prospective Multicenter Italian Endocarditis Study Cohort. Antimicrob Agents Chemother..

[27] Wang R, Li X, Wang Q, Zhang Y, Wang H (2017). Microbiological characteristics and clinical features of cardiac implantable electronic device infections at a tertiary hospital in China. Front Microbiol..

[28] Viola GM, Awan LL, Darouiche RO (2010). Nonstaphylococcal infections of cardiac implantable electronic devices. Circulation.

[29] Esquer Garrigos Z, George MP, Vijayvargiya P, Tan EM, Farid S, Abu Saleh OM, Friedman PA, Steckelberg JM, DeSimone DC, Wilson WR, Baddour LM, Sohail MR (2019). Clinical presentation, management, and outcomes of cardiovascular implantable electronic device infections due to Gram-negative versus Gram-positive bacteria. Mayo Clin Proc..

[30] Aleong RG, Zipse MM, Tompkins C, Aftab M, Varosy P, Sauer W, Kao D. Analysis of outcomes in 8304 patients undergoing lead extraction for infection. J Am Heart Assoc. 2020; 7; 9: e011473 10.1161/JAHA.118.011473 PMC7428595 32192410

[31] Lee DH, Gracely EJ, Aleem SY, Kutalek SP, Vielemeyer O (2015). Differences of mortality rates between pocket and nonpocket cardiovascular implantable electronic device infections. Pacing Clin Electrophysiol..

[32] Sohail MR, Henrikson CA, Braid-Forbes MJ, Forbes KF, Lerner DJ (2015). Increased long-term mortality in patients with cardiovascular implantable electronic device infections. Pacing Clin Electrophysiol..

[33] Le KY, Sohail MR, Friedman PA, Uslan DZ, Cha SS, Hayes DL, Wilson WR (2011). Clinical predictors of cardiovascular implantable electronic device-related infective endocarditis. Pacing Clin Electrophysiol.

[34] Tarakji KG, Wazni OM, Harb S, Hsu A, Saliba W, Wilkoff BL (2014). Risk factors for 1-year mortality among patients with cardiac implantable electronic device infection undergoing transvenous lead extraction: The impact of the infection type and the presence of vegetation on survival. Europace.

[35] Nishii N, Morimoto Y, Miyoshi A, Tsukuda S, Miyamoto M, Kawada S, Nakagawa K, Watanabe A, Nakamura K, Morita H, Morimatsu H, Kusano N, Kasahara S, Shoda M, Ito H (2019). Prognosis after lead extraction in patients with cardiac implantable electronic devices infection: Comparison of lead-related infective endocarditis with pocket infection in a Japanese single-center experience. J Arrhythm..

[36] Maytin M, Jones SO, Epstein LM (2012). Long-term mortality after transvenous lead extraction. Circ Arrhythm Electrophysiol..

[37] Habib A, Le KY, Baddour LM, Friedman PA, Hayes DL, Lohse CM, Wilson WR, Steckelberg JM, Sohail MR, Mayo Cardiovascular Infections Study Group (2013). Predictors of mortality in patients with cardiovascular implantable electronic device infections. Am J Cardiol..

