# Supplementary material for: Influence of the type of pathogen on the clinical course of infectious complications related to cardiac implantable electronic devices
Source: Sci Rep. 2021 Jul 21;11:14864. doi: 10.1038/s41598-021-94168-7 (PMC8295258; doi:10.1038/s41598-021-94168-7)
Supplement: Supplementary file 1 — Supplementary Tables. [file 41598_2021_94168_MOESM1_ESM.docx]

**Influence of the type of pathogen on the clinical course of infectious complications related to cardiac implantable electronic devices**

**Authors:** Anna Polewczyk^1,2^, Wojciech Jacheć^3^, Luca Segreti^4^, Maria Grazia Bongiorni ^4^, Andrzej Kutarski^5^

^1^ Collegium Medicum of Jan Kochanowski University, Kielce, Poland

^2^ Department of Cardiac Surgery, Świętokrzyskie Cardiology Center, Kielce, Poland

^3^ 2nd Department of Cardiology, Faculty of Medical Sciences in Zabrze, Medical University of Silesia, Poland

^4^ Department of Cardiology, University Hospital of Pisa, Italy

^5^ Department of Cardiology, Medical University, Lublin, Poland

Table S1 Combination of culture results in study population

|  | LRIE | PI |
| --- | --- | --- |
| Culture (all); n (%) | 773 (100) | 468 (100) |
| Culture positive; n (%) | 586 (75,81) | 245 (52,35) |
| Culture negative; n (%) | 187 (24,19) | 223 (47,65) |
| Vegetations (-), culture (-); n (%) | 41 (5,30) |  |
| Vegetations (-), culture (+); n (%) | 210 (27,17) |  |
| Vegetations (+), culture (-); n (%) | 148 (19,15) |  |
| Vegetations (+), culture (+); n (%) | 374 (48,38) |  |
| Staphylococcus aureus (all) | | |
| MSSA; n (%) | 180 (23,29) | 87 (18,59) |
| MRSA; n (%) | 14 (1,81) | 10 (2,14) |
| Staphylococcus epidermidis (all) | | |
| MSSE; n (%) | 141 (18,24) | 75 (16,03) |
| MRSE; n (%) | 78 (10,09) | 23 (4,91) |
| Other Staphylococci; (MRSA) / second bacteria | | |
| All; n (%); | 97 (12,55); (17) / 14 | 31 (6,62); (8) / 5 |
| S capitis; n | 18 (0) / 3 | 1 (0) / 0 |
| S hominis; n | 32 (6) / 3 | 10 (2) / 1 |
| S warneri; n | 12 (0) / 2 | 4 (1) / 0 |
| S auricularis; n | 6 (2) / 0 | 1 (0) / 0 |
| S lungdunesis ; n | 3 (0) / 0 | 2 (0) / 0 |
| S haemolyticus; n | 17 (5) / 2 | 7 (2) / 4 |
| S sppecies; n | 2 (0) / 1 | 0 (0) /0 |
| S Schleifleri; n | 1 (1) / 1 | 2 (0) / 0 |
| S bovis; n | 1 (0) / 0 | 0 (0) / 0 |
| S saprophiticus; n | 0 (0) / 0 | 1 (0) / 0 |
| S cohni; n | 1 (1) / 0 | 0 (0) / 0 |
| S simulans; n | 1 (1) / 0 | 1 (1) / 0 |
| S chromogenes; n | 2 (1) / 0 | 0 (0) / 0 |
| S mutans ; n | 1 (0) / 0 | 0 (0) / 0 |
| S xylosus; n | 0 (0) / 1 | 0 (0) / 0 |
| S salivarius; n | 0 (0) / 1 | 1 (1) / 0 |
| S lentus; n | 0 (0) / 0 | 1 (1) / 0 |
| MRSA / second staphylococcus; n | 0 (0) / 0 | 0 (0) / 0 |
| MRSA / second staphylococcus; n | 0 (0) / 0 | 0 (0) / 0 |
| Other staphylococcus (MRSA) / second staphylococcus; n | 0 (0) / 0 | 0 (0) / 0 |
| S. aureus + G (+) | 0 (0) | 0 (0) |
| S. aureus + G (-) | 4 (1) | 5 (0) |
| S,. epidermidis + G (+) | 1 (0) | 0 (0) |
| S. epidermidis + G (-) | 7 (1) | 1 (0) |
| Other S.+ G (+) | 2 (0) | 0 (0) |
| Other S.+ G (-) | 5 (1) | 0 (0) |
| G (+) + G (-) | 1 | 1 |
| G (-) + G (-) | 3 | 0 |
| Staphylococccus + Candida albicans | 0 (0) | 2 (0) |
| Other Gram-positive bacteria than staphylococcus / (second bacteria) | | |
| All; n (%); | 16 (2,07); (3) | 2 (0,43); (0) |
| Bacillus spp.; n | 1 (0) | 0 (0) |
| Brevibacterium casei; n | 1 (0) | 0 (0) |
| Kocuria kristinae; n | 1 (0) | 0 (0) |
| Micrococcus sp.; n | 3 (1) | 0 (0) |
| Propionebacterium acnes; n | 1 (1) | 0 (0) |
| Corynebacterium jeikeium (IS); n | 1 (0) | 0 (0) |
| Corynebacterium amycolat.; n | 2 (0) | 0 (0) |
| Corynebacterium species; n | 0 (0) | 1 (0) |
| Streptococcus mitis; n | 2 (0) | 0 (0) |
| Streptococcus sanguinis; n | 1 (0) | 0 (0) |
| Streptococcus galloticus; n | 1 (1) | 0 (0) |
| Streptococcus salivarius; n | 1 (0) | 1 (0) |
| Streptococcus agalatiae; n | 0 (0) | 0 (0) |
| Streptococcus viridans; n | 1 (0) | 0 (0) |
| Other Gram-negative/ (second bacteria) | | |
| All; n (%); | 54 (6,99); (20) | 24 (5,13); (7) |
| Brevundimonas vesicularis; n | 1 (1) | 0 (0) |
| Haemophilus influenzae; n | 1 (0) | 0 (0) |
| Serratia marcescens; n | 1 (0) | 0 (1) |
| Acinetobacter iwoffii; n | 2 (1) | 0 (0) |
| Acinetobacter baumanni; n | 4 (2) | 0 (1) |
| Acinetobacter beniamini; n | 0 (0) | 1 (0) |
| Citrobacter; n | 0 (0) | 1 (0) |
| Enterobacter cloacae; n | 6 (1) | 3 (0) |
| Proteus mirabilis; n | 5 (1) | 1 (0) |
| Proteus vulgaris; n | 1 (0) | 0 (0) |
| Klebsiella pneumonie; n | 9 (2) | 1 (0) |
| Klebsiella oxytoca; n | 0(0) | 0 (0) |
| Morganella morgani; n | 0 (0) | 3 (0) |
| Pseudomonas aeruginosa; n | 9 (2) | 3 (3) |
| Pseudomonas stutzeri; n | 1 (0) | 1 (0) |
| Escherichia coli; n | 15 (7) | 2 (0) |
| Enterococcus faecalis; n | 17 (3) | 1 (0) |
| Stenotrophomonas maltophilia; n | 2 (0) | 0 (1) |
| Finegoldia magna; n | 0 (0) | 0 (1) |
| Fungal infections / (second pathogen) | | |
| All; n (%); | 6 (0,78); (0) | 2 (0,43); (2) |
| Aspergillus fumigatus | 1 (0) | 0 (0) |
| Candida albicans | 5 (0) | 2 (2) |

Table S2 LRIE population- clinical and procedural data taking into account the type of pathogen

| **LRIE** | Culture negative | S aureus (all) | S epidermidis (all) | Other staphylococci | Other G positive | Other G negative | Candida / Aspargillus | ANOVA Kruskala Wallisa  P |
| --- | --- | --- | --- | --- | --- | --- | --- | --- |
|  | A | B | C | D | E | F | G |  |
| Blood culture n (%) | 187  (24,19) | 194  (25,10) | 219**^a^**  (28,33) | 97**^AAABBBCCC^**  (12,55) | 16 **^AAABBBCCCDDD^**  (2,07) | 54 **^AAABBBCCCDDDEEE^**  (6,99) | 6 **^AAABBBCCCDDDeFFF^**  (0,78) | P<0,001 |
| **Demographics** |  |  |  |  |  |  |  |  |
| Male  n (%) | 119  (36,36)  n=187 | 161 ^AAA^  (82,99)  n=194 | 159**^aB^**  (72,60)  n=219 | 68 **^B^**  (70,10)  n=97 | 11  (68,75)  n=16 | 37  (68,52)  n=54 | 4  (66,67)  n=6 | p=0,004 |
| Female  n (%) | 68  (36,36)  n=187 | 33 ^AAA^  (17,01)  n=194 | 60 **^aB^**  (27,40)  n=219 | 29 **^B^**  (29,90)  n=97 | 5  (31,25)  n=16 | 17  (31,48)  n=54 | 2  (33,33)  n=6 | p=0,020 |
| Mean age (first implantation)  mean, sd | 59,428  ±14,576  n=187 | 63,902 ^A^  ±13,664  n=194 | 58,973**^BBB^**  ±14,707  n=219 | 58,320**^BB^**  ±15,184  n=97 | 60,063  ±17,510  n=16 | 61,352  ±12,442  n=54 | 57,667  ±33,170  n=6 | p=0,007 |
| Mean age (TLE)  mean, sd | 67,380  ±12,957  n=187 | 70,170  ±12,366  n=194 | 66,712  ±13,565  n=219 | 66,495  ±14,340  n=97 | 66,563  ±15,214  n=16 | 68,259  ±11,768  n=54 | 62,833  ±31,410  n=6 | p=0,133 |
| BMI [kg / m^2^]  mean, sd | 27,235  ±4,077  n=176 | 26,999  ±4,631  n=164 | 27,595  ±4,375  n=208 | 26,797  ±3,849  n=93 | 26,465  ±3,754  n=16 | 27,385  ±3,782  n=49 | 25,374  ±5,420  n=5 | p=0,544 |
| NYHA class  mean, sd | 1,715  ±0,757  n=186 | 1,670  ±0,778  n=194 | 1,739  ±0,826  n=218 | 1,629  ±0,712  n=97 | 1,625  ±0,719  n=16 | 1,796  ±0,762  n=54 | 1,333  ±0,516  n=6 | p=0,337 |
| LVEF  mean, sd | 48,503  ±15,455  n=185 | 47,125  ±13,764  n=184 | 48,855  ±14,013  n=214 | 49,916  ±12,748  n=95 | 44,000  ±15,446  n=15 | 44,692  ±15,004  n=52 | 45,500  ±9,138  n=6 | p=0,126 |
| Valvular implants  n (%) | 12  (6,42)  n=187 | 13  (6,70)  n=194 | 13  (5,94)  n=219 | 2  (2,06)  n=97 | 1  (6,25)  n=16 | 5  (9,26)  n=54 | 0  (0,00)  n=6 | p=0,632 |
| Prior sternotomy  n (%) | 36  (19,25)  n=187 | 24  (12,37)  n=194 | 29  (13,24)  n=219 | 16**^BB^**  (16,49)  n=97 | 1  (6,25)  n=16 | 14  (25,93)  n=54 | 2  (33,33)  n=6 | p=0,084 |
| Hypertension  n (%) | 124  (66,31)  n=187 | 128  (65,98)  n=194 | 136  (62,10)  n=219 | 59  (60,82)  n=97 | 8  (50,00)  n=16 | 28  (51,85)  n=54 | 3  (50,00)  n=6 | p=0,503 |
| Diabetes  n (%) | 45  (24,19)  n=186 | 59  (30,89)  n=191 | 50**^b^**  (23,36)  n=214 | 12**^AB^**  (12,37)  n=97 | 3  (18,75)  n=16 | 12  (23,08)  n=52 | 1  (16,67)  n=6 | p=0,048 |
| History of CKD or eGFR <60 ml/min/1.73 m^2^  n (%) | 8  (4,49)  n=178 | 22 ^AA^  (12,50)  n=176 | 4**^BBB^**  (1,91)  n=209 | 2**^BB^**  (2,20)  n=91 | 1  (7,14)  n=14 | 1  (2,00)  n=50 | 0  (0,00)  n=6 | p<0,001 |
| Permanent atrial fibrillation  n (%) | 36  (19,35)  n=186 | 45  (23,68)  n=190 | 40  (18,60)  n=215 | 22  (22,68)  n=97 | 5  (31,25)  n=16 | 17  (32,08)  n=53 | 1  (16,67)  n=6 | p=0,359 |
| Permanent anticoagulation  n (%) | 52  (28,42)  n=183 | 62  (33,33)  n=186 | 71  (33,18)  n=214 | 29  (30,53)  n=95 | 6  (37,50)  n=16 | 27**^AABCD^**  (51,92)  n=52 | 1  (16,67)  n=6 | p=0,079 |
| Permanent antiplatelet therapy  n (%) | 81  (44,26)  n=183 | 68  (36,56)  n=186 | 78  (36,45)  n=214 | 45  (47,37)  n=95 | 8  (50,00)  n=16 | 23  (44,23)  n=52 | 1  (16,67)  n=6 | p=0,245 |
| **Preoperative pacing system information** |  |  |  |  |  |  |  |  |
| Device with ICD lead  n (%) | 68  (36,36)  n=187 | 72  (37,11)  n=194 | 74  (33,79)  n=219 | 32  (32,99)  n=97 | 4  (25,00)  n=16 | 23  (42,59)  n=54 | 1  (16,67)  n=6 | p=0,715 |
| Dwelling time for oldest lead (months) mean ± sd | 96,444  ±77,717  n=187 | 75,954**^AA^**  ±70,027  n=194 | 93,740**^BBB^**  ±65,033  n=219 | 98,711**^BB^**  ±67,548  n=97 | 78,688  ±57,747  n=16 | 83,111  ±60,025  n=54 | 62,500  ±46,980  n=6 | p=0,003 |
| Number of all operative procedures before TLE (implantations, reimplantation, upgrading, pocket revision) mean, sd | 2,251  ±1,490  n=187 | 2,309  ±1,786  n=194 | 2,785**^AAABBB^**  ±1,778  n=219 | 2,515**^b^**  ±1,602  n=97 | 2,125  ±1,500  n=16 | 2,074**^CC^**  ±1,163  n=54 | 1,500**^C^**  ±0,548  n=6 | p=0,006 |
| Lead abrasion  n (%) | 47  (32,64)  n=144 | 31  (31,00)  n=100 | 57  (32,57)  n=175 | 19  (24,05)  n=79 | 3  (23,08)  n=13 | 6  (15,00)  n=40 | 0  (0,00)  n=4 | p=0,191 |
| Vegetations presence  n (%) | 148  (79,14)  n=187 | 130**^AA^**  (67,01)  n=194 | 133**^AAA^**  (60,73)  n=219 | 62**^AA^**  (63,92)  n=97 | 10  (62,50)  n=16 | 35**^A^**  (64,81)  n=54 | 4  (66,67)  n=6 | p=0,012 |
| Presence of large vegetation >2cm2 / vegetation presence / infective endocarditis n (%) | 36  (19,25)  n=187 | 32  (16,49)  n=194 | 43  (19,63)  n=219 | 16  (16,49)  n=97 | 7  (43,75)  n=16 | 11  (20,37)  n=54 | 1  (16,67)  n=6 | p=0,258 |
| **TLE procedure** |  |  |  |  |  |  |  |  |
| Number of leads in system  mean ± sd | 2,005  ±0,684  n=187 | 1,959  ±0,733  n=194 | 1,991  ±0,621  n=219 | 1,814**^AA^**  ±0,565  n=97 | 2,063  ±0,574  n=16 | 1,815  ±0,746  n=54 | 1,333**^CdE^**  ±0,516  n=6 | p=0,035 |
| Total number of leads in patient  mean ± sd | 2,219  ±0,836  n=187 | 2,186  ±0,887  n=194 | 2,379  ±0,967  n=219 | 2,052  ±0,698  n=97 | 2,250  ±0,856  n=16 | 1,981**^C^**  ±0,869  n=54 | 1,667**^C^**  ±0,837  n=6 | p=0,009 |
| Mean number of abandoned leads in patients mean ± sd | 0,2139  ±0,550  n=187 | 0,227  ±0,585  n=194 | 0,388  ±0,749  n=219 | 0,237  ±0,541  n=97 | 0,188  ±0,544  n=16 | 0,185  ±0,517  n=54 | 0,333**^d^**  ±0,516  n=6 | p=0,219 |
| Technical problems or complications during TLE number of pts  n (%) | 32  (17,11)  n=187 | 26  (13,40)  n=194 | 35  (15,98)  n=219 | 13  (13,40)  n=97 | 1  (6,25)  n=16 | 5  (9,26)  n=54 | 0  (0,00)  n=6 | p=0,570 |
| Procedure duration time [min]  mean ± sd | 106,882  ±50,521  n=186 | 105,568  ±61,055  n=192 | 114,780**^B^**  ±56,627  n=218 | 107,375  ±48,474  n=96 | 112,938  ±47,096  n=16 | 94,434**^C^**  ±36,475  n=53 | 84,167  ±36,389  n=6 | p=0,068 |
| Periprocedural deaths  n (%) | 2  (1,07)  n=187 | 1  (0,52)  n=194 | 3  (1,37)  n=219 | 0  (0,00)  n=97 | 0  (0,00)  n=16 | 0  (0,00)  n=54 | 0  (0,00)  n=6 | p=0,842 |
| Full procedural success  n (%) | 174  (93,05)  n=187 | 186  (95,88)  n=194 | 205  (93,61)  n=219 | 94  (96,91)  n=97 | 16  (100,00)  n=16 | 51  (94,44)  n=54 | 6  (100,00)  n=6 | p=0,644 |
| Clinical success  n (%) | 180  (96,26)  n=187 | 188  (96,91)  n=194 | 211  (96,35)  n=219 | 95  (97,94)  n=97 | 16  (100,00)  n=16 | 53  (98,15)  n=54 | 6  (100,00)  n=6 | p=0,937 |
| Major complications n (%) | 4  (2,14)  n=187 | 5  (2,58)  n=194 | 4  (1,83)  n=219 | 1  (1,03)  n=97 | 0  (0,00)  n=16 | 0  (0,00)  n=54 | 0  (0,00)  n=6 | p-0,870 |
| Minor complications  n (%) | 11  (5,88)  n=187 | 11  (5,67)  n=194 | 14  (6,39)  n=219 | 4  (4,12)  n=97 | 3  (18,75)  n=16 | 5  (9,26)  n=54 | 0  (0,00)  n=6 | p=0,375 |
| Periprocedural deaths  n (%) | 2  (1,07)  n=187 | 1  (0,52)  n=194 | 3  (1,37)  n=219 | 0  (0,00)  n=97 | 0  (0,00)  n=16 | 0  (0,00)  n=54 | 0  (0,00)  n=6 | p=0,842 |
| Time of follow-up [days]  mean ± sd | 1368,3  ±1024,8  N=172 | 1326,4**^a^**  ±1028,1  N=154 | 1867,4**^AABBB^**  ±1044,0  N=201 | 1644,5**^BC^** ±945,9  N=89 | 1503,8  ±1265,8  N=16 | 1337,5**^CCCd^**  ±948,0  N=48 | 1291,2  ±450,0  N=5 | P=0,003 |
| Death during follow-up n (%) | 56  (32,56)  n=172 | 60  (38,96)  n=154 | 69  (34,33)  n=201 | 35  (39,33)  n=89 | 10**^AAABCd^**  (62,50)  n=16 | 21**^a^**  (43,75)  n=48 | 3  (60,00)  n=56 | p=0,051 |

^abcdef^ – p > 0,05 and <0,1; ^ABCDEF^ – p< 0,05; ^AABBCCDDEEFF^ – p< 0,01; ^AAABBBCCCDDDEEEFFF^ – p< 0,001

^a^ p <0,1; ^A^ p<0,05; ^AA^ p<0,01; ^AAA^ p<0,001 when compared to A

^b^ p <0,1; ^B^ p<0,05; ^BB^ p<0,01; ^BBB^ p<0,001 when compared to B

^c^ p <0,1; ^C^ p<0,05; ^CC^ p<0,01; ^CCC^ p<0,001 when compared to C

^d^ p <0,1; ^D^ p<0,05; ^DD^ p<0,01; ^DDD^ p<0,001 when compared to D

^e^ p <0,1; ^E^ p<0,05; ^EE^ p<0,01; ^EEE^ p<0,001 when compared to E

AAI – atrial chamber pacemaker, AF – atrial fibrillation, BP – bipolar; CKD - chronic kidney disease; CRT-D - cardiac resynchronisation therapy defibrillator; CRT-P - cardiac resynchronization pacemaker; D – dual chamber; DDD – dual chamber pacemaker, eGFR - glomerular filtration rate; ICD - implantable cardioverter defibrillator; LRIE- lead reated infective endocarditis; VEF -left ventricular ejection fraction, PM – pacing; SD – standard deviation, TLE - transvenous lead extraction; V – ventricular; VDD - single-lead dual sensing pacing; VVI – ventricular chamber pacemaker; UP – unipolar

Table S3 PI population: clinical and procedural data taking into account the type of pathogen

| PI | Culture negative | Staphylococcus aureus  (all) | Staphylococcus epidermidis (all) | Other staphylococcus  (all) | Other than staphylococcus G (+) bacteria | G (-) bacteria | Candida albicans | ANOVA Kruskala Wallisa  P |
| --- | --- | --- | --- | --- | --- | --- | --- | --- |
|  | **A** | **B** | **C** | **D** | **E** | **F** | G |  |
| Number n (%) | 223  (47,65) | 97**^AAA^**  (20,73) | 98 **^AAA^**  (20,94) | 31**^AAABBBCCC^**  (6,62) | 2 **^AAABBBCCCDDD^**  (0,43) | 17**^AAABBBCCCDDDEE^**  (3,63) | 2 **^AAABBBCCCDDDFF^**  (0,43) |  |
| **Demographics** |  |  |  |  |  |  |  |  |
| Male  n (%) | 160  (71,75)  n=223 | 81**^A^**  (83,51)  n=97 | 75  (76,53)  n=98 | 24  (77,42)  n=31 | 1  (50,00)  n=2 | 6**^ABBBd^**  (35,29)  n=17 | 1  (50,00)  n=2 | p=0,026 |
| Female  n (%) | 63  (28,25)  n=223 | 16  (16,49)  n=97 | 23  (23,47)  n=98 | 7  (22,58)  n=31 | 1  (50,00)  n=2 | 11**^ABBBd^**  (64,71)  n=17 | 1  (50,00)  n=2 | p=0,026 |
| Mean age (first implantation) [years]  mean, sd | 60,112  ±15,064  n=223 | 62,340  ±14,883  n=97 | 62,490  ±15,507  n=98 | 59,323^Bc^  ±17,213  n=31 | 80,000**^AD^**  ±4,243  n=2 | 67,294**^a^**  ±14,008  n=17 | 57,000  ±9,899  n=2 | p=0,049 |
| Mean age (TLE) [years]  mean, sd | 67,655  ±13,525  n=223 | 68,691  ±14,346  n=97 | 68,673  ±14,510  n=98 | 65,742  ±16,160  n=31 | 83,000  ±2,828  n=2 | 70,647  ±13,138  n=17 | 60,000  ±9,899  n=2 | p=0,315 |
| BMI [kg/m^2^] | 27,081  ±3,435  n=188 | 25,976  ±3,958  n=74 | 27,164  ±3,529  n=84 | 28,096  ±3,068  n=26 | 23,788  ±5,581  n=2 | 26,496  ±3,736  n=15 | 23,629  ±5,857  n=2 | p=0,164 |
| NYHA class  mean ± sd | 1,610  ±0,726  n=223 | 1,526  ±0,682  n=97 | 1,602  ±0,714  n=98 | 1,355**^BC^**  ±0,551  n=31 | 3,000**^AD^**  ±0,000  n=2 | 1,438**^D^**  ±0,629  n=17 | 1,000  ±0,000  n=2 | p=0,068 |
| LVEF [%]  mean, sd | 47,895  ±14,178  n=209 | 48,318  ±13,359  n=88 | 47,989  ±15,534  n=94 | 50,433  ±12,514  n=30 | 40,000  ±28,284  n=2 | 45,647  ±14,996  n=17 | 60,000  ±0,000  n=2 | p=0,852 |
| Valvular implants  n (%) | 13  (5,83)  n=223 | 7  (7,22)  n=97 | 5  (5,10)  n=98 | 1  (3,23)  n=31 | 0  (0,00)  n=2 | 3  (17,65)  n=17 | 0  (0,00)  n=2 | p=0,434 |
| Prior sternotomy  n (%) | 30  (13,51)  n=223 | 19  (19,59)  n=97 | 14  (14,43)  n=98 | 2  (6,45)  n=31 | 0  (0,00)  n=2 | 7**^AAbD^**  (41,18)  n=17 | 0  (0,00)  n=2 | p=0,025 |
| Arterial hypertension  n (%) | 137  (59,83)  n=223 | 58  (61,70)  n=97 | 62  (65,26)  n=98 | 19  (38,00)  n=31 | 2  (100,00)  n=2 | 10  (37,04)  n=17 | 1  (50,00)  n=2 | p=0,898 |
| Diabetes  n (%) | 42  (19,00)  n=221 | 15  (15,63)  n=96 | 17  (17,35)  n=98 | 9  (29,03)  n=31 | 0  (0,00)  n=2 | 5  (29,41)  n=17 | 1  (50,00)  n=2 | p=0,469 |
| History of CKD or eGFR  <60 ml/min/1.73 m^2^  n (%) | 19  (9,69)  n=196 | 6  (7,23)  n=83 | 6  (6,82)  n=88 | 3  (10,00)  n=30 | 0  (0,00)  n=2 | 2  (14,29)  n=14 | 1  (50,00)  n=2 | p=0,894 |
| Permanent atrial fibrillation  n (%) | 58  (26,13)  n=222 | 30  (30,93)  n=97 | 24  (24,74)  n=97 | 5  (16,13)  n=31 | 0  (0,00)  n=2 | 8  (47,06)  n=17 | 0  (0,00)  n=2 | p=0,207 |
| Permanent anticoagulation  n (%) | 71  (33,02)  n=215 | 29  (31,52)  n=92 | 31  (33,70)  n=92 | 7  (22,58)  n=31 | 1  (50,00)  n=2 | 5  (31,25)  n=16 | 0  (0,00)  n=2 | P=0,878 |
| Antiplatelet therapy  n (%) | 87  (40,47)  n=215 | 31  (33,70)  n=92 | 39  (42,39)  n=92 | 7  (22,58)  n=31 | 1  (50,00)  n=2 | 7  (43,75)  n=16 | 1  (50,00)  n=2 | p=0,364 |
| **Preoperative pacing system information** |  |  |  |  |  |  |  |  |
| Device with ICD lead  n (%) | 80  (35,87)  n=223 | 43  (44,33)  n=97 | 38  (38,78)  n=98 | 6  (19,35)  n=31 | 1  (50,00)  n=2 | 5  (29,41)  n=17 | 0  (0,00)  n=2 | p=0,207 |
| Dwelling time for oldest lead  months (mean, sd) | 91,395  ±75,870  n=223 | 77,021  ±65,037  n=97 | 74,888  ±59,611  n=98 | 78,032  ±52,940  n=31 | 33,500  ±19,092  n=2 | 41,412**^AAADD^**  ±44,626  n=17 | 34,500  ±4,950 | p=0,008 |
| Number of all operative procedures before TLE (implantations, reimplantation, upgrading, pocket revision) mean ± sd | 2,372  ±1,471  n=223 | 2,474  ±1,385  n=97 | 2,418  ±1,377  n=98 | 2,419  ±0,992  n=31 | 2,500  ±2,121  n=2 | 1,765  ±0,831  n=17 | 2,000  ±1,414 | p=0,390 |
| Lead abrasion  n (%) | 23  (21,70)  n=106 | 9  (25,00)  n=36 | 11  (18,64)  n=59 | 6  (27,27)  n=22 | 0  (0,00)  n=2 | 1  (9,09)  n=11 | 2  (100,00)  n=2 | p=0,770 |
| **TLE procedure** |  |  |  |  |  |  |  |  |
| Number of leads in system  mean ± sd | 1,996  ±0,713  n=223 | 2,052  ±0,667  n=97 | 1,908  ±0,644  n=98 | 1,935  ±0,512  n=31 | 1,000  ±0,000  n=2 | 1,706  ±0,686  n=17 | 1,500  ±0,707  n=2 | p=0,096 |
| Total number of leads in patient  mean ± sd | 2,197  ±0,859  n=223 | 2,371  ±0,809  n=97 | 2,153**^b^**  ±0,745  n=98 | 2,226**^BC^**  ±0,735  n=31 | 1,000**^AD^**  ±0,000  n=2 | 1,765**^aBB^**  ±0,664  n=17 | 2,500  ±0,707  n=2 | p=0,010 |
| Number of patients with abandoned leads  n (%) | 33  (14,80)  n=223 | 23^a^  (23,71)  n=97 | 20  (20,41)  n=98 | 7  (22,58)  n=31 | 0  (0,00)  n=2 | 1  (5,88)  n=17 | 1  (50,00)  n=2 | P=0,248 |
| Mean number of abandoned leads in patients mean ± sd | 0,201  ±0,598  n=223 | 0,319  ±0,754  n=97 | 0,245  ±0,664  n=98 | 0,290  ±0,588  n=31 | 0,000  ±0,000  n=2 | 0,059  ±0,243  n=17 | 1,000  ±1,414  n=2 | p=0,255 |
| Procedure duration time [min]  mean ±sd | 102,448  ±45,193  n=223 | 103,13  ±745,713  n=97 | 98,052  ±43,526  n=98 | 99,065  ±41,581  n=31 | 100,000  ±14,142  n=2 | 86,765  ±38,199  n=17 | 102,500  ±3,536  n=2 | p=0,827 |
| Full procedural success  n (%) | 208  (93,27)  n=223 | 92  (94,85)  n=97 | 93  (94,90)  n=98 | 30  (96,77)  n=31 | 2  (100,00)  n=2 | 16  (94,12)  n=17 | 2  (100,000)  n=2 | p=0,962 |
| Clinical success  n (%) | 214  (95,96)  n=223 | 92  (94,85)  n=97 | 95  (96,94)  n=98 | 31  (100,00)  n=31 | 2  (100,00)  n=2 | 16  (94,12)  n=17 | 2  (100,000)  n=2 | p=0,829 |
| Major complications  n (%) | 1  (0,45)  n=223 | 2  (2,06)  n=97 | 0  (0,00)  n=98 | 0  (0,00)  n=31 | 0  (0,00)  n=2 | 1  (5,88)  n=17 | 0  (0,00)  n=2 | p=0,141 |
| Minor complications  n (%) | 8  (3,59)  n=223 | 6  (6,19)  n=97 | 4  (4,08)  n=98 | 1  (3,23)  n=31 | 0  (0,00)  n=2 | 2  (11,76)  n=17 | 0  (0,00)  n=2 | p=0,636 |
| Follow-up [days]  mean ± sd | 1608,9  ±953,4  n=170 | 1615,1  ±1163,1  n=75 | 1774,5  ±969,5  n=84 | 2091,1**^Ab^**  ±1221,3  n=30 | 407,00**^aCd^**  ±26,87  n=2 | 1430,5**^d^**  ±1077,1  n=14 | 602,000  ±374,767  n=2 | p=0,067 |
| Death during follow-up  n (%) | 50  (29,41)  n=170 | 26  (34,67)  n=75 | 25  (29,76)  n=84 | 4**^aBc^**  (13,33)  n=30 | 2**^AbCDD^**  (100,0)  n=2 | 8**^ACD^**  (57,14)  n=15 | 0  (0,00)  n=2 | p=0,015 |

^abcdef^ – p > 0,05 and <0,1; ^ABCDEF^ – p< 0,05; ^AABBCCDDEEFF^ – p< 0,01; ^AAABBBCCCDDDEEEFFF^ – p< 0,001

AAI – atrial chamber pacemaker, AF – atrial fibrillation, BP – bipolar; CKD - chronic kidney disease; CRT-D - cardiac resynchronisation therapy defibrillator; CRT-P - cardiac resynchronization pacemaker; D – dual chamber; DDD – dual chamber pacemaker, eGFR - glomerular filtration rate; ICD - implantable cardioverter defibrillator; LVEF -left ventricular ejection fraction, PM – pacing; SD – standard deviation, TLE - transvenous lead extraction; V – ventricular; VDD - single-lead dual sensing pacing; VVI – ventricular chamber pacemaker; UP – unipolar
